# Ena orchestrates remodelling within the actin cytoskeleton to drive robust *Drosophila* macrophage chemotaxis

**DOI:** 10.1242/jcs.224618

**Published:** 2019-02-18

**Authors:** Andrew J. Davidson, Tom H. Millard, Iwan R. Evans, Will Wood

**Affiliations:** 1 School of Cellular and Molecular Medicine, Faculty of Biomedical Sciences, University of Bristol, Bristol BS8 1TD, UK; 2 Faculty of Biology, Medicine and Health, University of Manchester, Michael Smith Building, Oxford Road, Manchester M13 9PT, UK; 3 Department of Infection, Immunity and Cardiovascular Disease, University of Sheffield, Sheffield S10 2RX, UK; 4 The Bateson Centre, University of Sheffield, Sheffield S10 2TN, UK

**Keywords:** *Drosophila*, Ena, Actin, Hemocyte, Macrophage, Migration

## Abstract

The actin cytoskeleton is the engine that powers the inflammatory chemotaxis of immune cells to sites of tissue damage or infection. Here, we combine genetics with live *in vivo* imaging to investigate how cytoskeletal rearrangements drive macrophage recruitment to wounds in *Drosophila*. We find that the actin-regulatory protein Ena is a master regulator of lamellipodial dynamics in migrating macrophages, where it remodels the cytoskeleton to form linear filaments that can then be bundled together by the cross-linker Fascin (also known as Singed in flies). In contrast, the formin Dia generates rare, probing filopods for specialised functions that are not required for migration. The role of Ena in lamellipodial bundling is so fundamental that its overexpression increases bundling even in the absence of Fascin by marshalling the remaining cross-linking proteins to compensate. This reorganisation of the lamellipod generates cytoskeletal struts that push against the membrane to drive leading edge advancement and boost cell speed. Thus, Ena-mediated remodelling extracts the most from the cytoskeleton to power robust macrophage chemotaxis during their inflammatory recruitment to wounds.

## Abstract (precis)

**Summary:** Macrophages must migrate to a variety of stimuli, including inflammatory wounds. We identify the actin-regulatory protein Ena as a master remodeller of the cytoskeleton within migrating macrophages *in vivo*.

## INTRODUCTION

Cell migration is crucial to whole swathes of fundamental biology, including embryogenesis, cancer metastasis, wound healing and immunity. This is perhaps most evident in immune cells, such as macrophages, which are required to rapidly migrate to sites of damage and infection (Wood and Martin, 2017). Through chemotaxis, immune cells are drawn towards wounds by detecting and migrating towards signals released by damaged tissue. For example, both fly and fish leukocytes rapidly respond to the early damage signal, H_2_O_2_, detected via Src family kinases (Niethammer et al., 2009; Moreira et al., 2010; Yoo et al., 2011; Razzell et al., 2013).

The cellular protrusions that underlie motility are formed through rearrangements in the actin cytoskeleton enacted through the activity of highly conserved actin regulators. For example, the Arp2/3 complex creates dendritic networks of F-actin driving the extension of the lamellipod during cell motility (Mullins et al., 1998; Svitkina and Borisy, 1999). The spatial and temporal activity of the Arp2/3 complex is controlled by activators such as WASP, SCAR/WAVE and WASH (Machesky and Insall, 1998; Machesky et al., 1999; Linardopoulou et al., 2007). For example, SCAR recruits the Arp2/3 complex to promote the formation of lamellipods. In contrast, other nucleators such as Ena/VASP family proteins and formins generate unbranched, linear filaments that can be bundled together (Pruyne et al., 2002; Breitsprecher et al., 2008). These bundles of F-actin are found within the lamellipod or projecting out of the cell as filopods (Svitkina et al., 2003). Ultimately a subset of actin regulators are collectively deployed to drive the extension of a certain type of protrusion, which in turn promotes a specific cellular behaviour, such as chemotaxis.

Increasingly sophisticated biochemical approaches and chemotactic chambers are advancing our understanding of *in vitro* cell migration (Vignjevic et al., 2003; Lee et al., 2010; Muinonen-Martin et al., 2010; Reymann et al., 2010; Wu et al., 2012). However, our ability to apply these findings to an *in vivo* setting lags behind due to the difficulty of studying cells within the context of a living tissue.

Here, we have utilised *Drosophila* embryonic macrophages as a model of actin dynamics, combining the powerful genetics of the fly, with the excellent live *in vivo* imaging possible in the embryo (Evans et al., 2010). Like their mammalian counterparts, these macrophages chemotax towards a wide range of stimuli, including bacterial infection and tissue damage through the extension of actin-rich protrusions (Wood and Jacinto, 2007).

Here, we demonstrate that Ena rather than the formin Dia is operating to organise actin within the lamellipod into Fascin-decorated bundles (Fascin is also known as Singed in flies). Ena is such a potent remodeller within the lamellipod that its overexpression can even compensate for the loss of bundlers such as Fascin. Through these bundles, Ena acts to reinforce the lamellipod and drive the leading edge forward, and thus underlies robust macrophage motility during the inflammatory response. Our findings demonstrate that Ena is a master regulator of the actin cytoskeleton within chemotaxing macrophages *in vivo*, ensuring the swiftest possible response to tissue damage and infection.

## RESULTS AND DISCUSSION

### Ena, rather than Dia, organises F-actin into linear bundles within the lamellipod

**Fig. 1.**
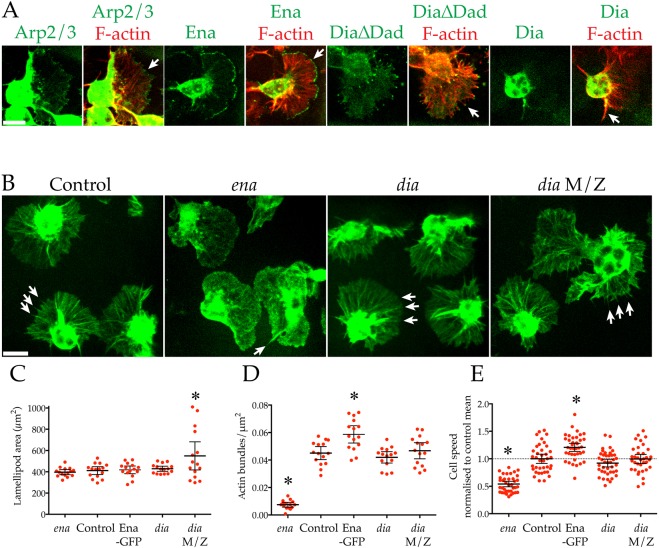
**Ena, but not Dia, is required for nearly all lamellipodial bundling and for efficient macrophage migration.** (A) Live, *in vivo* imaging of F-actin (LifeAct–mCherry, red) and key, GFP-tagged actin regulators (green, arrows) within macrophage lamellipods. Scale bar: 10 µm. (B) Control, *ena* and *dia* (*dia[2]* and *dia[2]*/*dia[5]* M/Z) macrophages expressing LifeAct–GFP. Loss of *ena* (but not *dia*) results in loss of almost all lamellipodial bundles (arrows). Scale bar: 10 µm. (C–E) Quantification of motility in control, *ena* and *dia* (*dia[2]* and *dia*[2]/*dia[5]* M/Z) mutants and macrophages overexpressing Ena–GFP. (C) Lamellipodial area (*ena*=396.57±11.04, control=411.68±17.20, Ena=418.33±17.16, *dia*=428.79±11.41, *dia* M/Z=549.77±61.64 µm^2^, mean±s.e.m., *n*=15 cells/genotype), (D) actin bundle density (*ena*=0.008±0.001, control=0.045±0.002, Ena=0.059±0.003, *dia*=0.042±0.002, *dia* M/Z=0.047±0.003 bundles/µm^2^, mean±s.e.m., *n*=15 cells/genotype) and (E) basal cell speed normalised to control mean (dashed line, *ena*=0.54±0.025, control=1.0±0.042, Ena=1.21±0.037, *dia*=0.92±0.034, *dia* M/Z=1.0±0.041 mean±s.e.m., *n*≥35 cells/genotype). Error bars are 95% c.i. *P<0.05 vs control mean (ANOVA).

The actin within the lamellipods of *Drosophila* embryonic macrophages is highly organised and is arranged into linear bundles. We sought to understand how these lamellipodial bundles are formed and how they contribute to macrophage chemotaxis. Live, *in vivo* imaging revealed that both GFP-tagged Arp2/3 complex and Ena–GFP localise to the leading edge of the lamellipod where the latter interacts with the tips of the lamellipodial actin bundles (Fig. 1A; Tucker et al., 2011). Although not as smoothly localised to the lamellipod edge as Ena, DiaΔDad–GFP (a constitutively active, truncated Dia commonly used as a probe) also localises to the tips of actin bundles (Fig. 1A; Homem and Peifer, 2009, Bilancia et al., 2014). However, DiaΔDad–GFP severely disrupted the architecture of the lamellipod and significantly reduced lamellipodial bundle number compared to control cells (Fig. S1A,B). In contrast to DiaΔDad–GFP, full-length Dia–GFP is seldom utilised as a probe due to its poor localisation, and we likewise found it to be predominantly cytosolic (Homem and Peifer, 2008). However, in a rare few examples, full-length Dia–GFP localised to the entire length of an individual actin bundle (Fig. 1A, Movie 1; Davis et al., 2015). As a constitutively active fragment of Dia, the increased activity of DiaΔDad–GFP is unsurprising. However, the different localisations of Dia–GFP versus DiaΔDad–GFP was concerning.

To distinguish the roles of Dia and Ena within the lamellipod, we visualised the actin cytoskeleton of *ena* and *dia* mutant macrophages (Fig. 1B; Movie 2). Ena is not required to extend lamellipods, in contrast to *scar* and *arp3* (subunit of the Arp2/3 complex) mutants (Fig. 1C; Fig. S1C,D; Evans et al., 2013). However, as we have previously shown, *ena* mutants had a near total loss of lamellipodial bundles, which correlated with a decrease in basal motility (Fig. 1B,D,E; Movie 2; Tucker et al., 2011). Conversely, as previously shown, Ena–GFP expression increases lamellipodial bundling and basal cell speed (Fig. 1D,E; Tucker et al., 2011). In contrast, no significant difference in macrophage basal motility was detected in either of two *dia* mutants (Fig. 1E). In the more severe, maternally zygotic *dia* (*dia[2]*/*dia[5]* M/Z) mutant, many macrophages were significantly larger (Fig. 1B,C) and were likely multinucleate (Castrillon and Wasserman, 1994). Importantly, when normalised to lamellipod area, neither *dia* mutant exhibited any significant difference in bundle number compared to controls (Fig. 1D). Furthermore, Dia–GFP localised to the residual lamellipodial bundles found in *ena* mutant macrophages (Fig. S1E). These findings are consistent with the localisation of full-length Dia–GFP to only a rare subset of actin bundles involved in specialised roles such as contact-induced repulsion (Davis et al., 2015).

In summary, lamellipodial bundling is required for robust immune cell motility. However, exactly how Ena increases bundle formation and how these bundles contribute to cell migration remained an open question we next sought to answer.

### Ena remodels actin within the lamellipod into Fascin cross-linked bundles

**Fig. 2.**
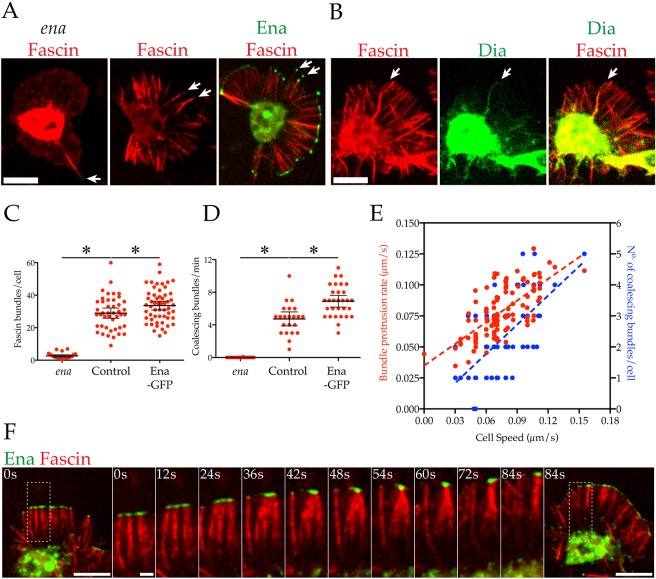
**Ena acts to generate and coalesce Fascin-decorated bundles within the lamellipod.** (A,B) Fascin–mCherry (red) expressed (A) in *ena*/control macrophages with or without Ena–GFP or (B) with Dia–GFP (green). Arrows highlight distinct Fascin bundles. Scale bars: 10 µm. (C,D) Fascin-mediated bundling in *ena*/control macrophages with or without Ena–GFP. (C) Number of Fascin-decorated bundles/cell (*ena*=2.645±0.33, control=28.93±1.55, Ena=33.58± 1.35 bundes/cell, mean±s.e.m., *n*>25 cells/genotype). (D) Number of bundle coalescence events/min (*ena*=0.01±0.01, control=4.73±0.41, Ena=6.90±0.35 bundles/cell, mean±s.e.m., *n*≥20 cells/genotype). Error bars are 95% c.i. *P<0.05 vs control mean (ANOVA). (E) Control basal cell speed correlates positively with individual bundle protrusion rates (red, r^2^=0.49, *n*=109) and number of coalescing bundles/cell (blue, r^2^=0.39, *n*=88). Both slopes are significantly different from zero (*P*<0.0001). (F) Sequence showing coalescing Ena (green)-capped, Fascin (red)-decorated bundles. The area of the dashed box is expanded in intervening panels. Time: seconds. Scale bars: 10 µm (end panels), 2 µm intervening panels.

Ena remodels branched actin within the lamellipod into linear bundles. Purified Ena can bundle F-actin *in vitro* (Bachmann et al., 1999; Schirenbeck et al., 2006)*.* However, within the lamellipod, Ena is confined to the leading edge and therefore cannot be directly responsible for bundling actin filaments (Fig. 1A, Rottner et al., 1999; Tucker et al., 2011). Instead, *in vivo* Ena co-operates with actin cross-linkers (Winkelman et al., 2014), and we found in *Drosophila* macrophages that Ena-capped lamellipodial bundles were indeed decorated with one such bundler, Fascin (Fig. 2A; Movie 3). Fascin also colocalised with Dia–GFP at the rare bundles that were positive for the latter (Fig. 2B).

Loss of *ena* resulted in a near total loss of Fascin bundles (Fig. 2A,C). Conversely, overexpression of Ena significantly increased Fascin bundle number. By following individual Fascin-decorated and Ena-capped bundles, we observed bundle coalescence within advancing lamellipods (Fig. 2F; Movie 3). This process is initiated when Ena-capped Fascin-labelled bundles contact one another. Once joined via their Ena caps, the Fascin-decorated bundles proceed to coalesce from the Ena caps downwards in a zipper-like manner. Furthermore, overexpression of Ena significantly increased the number of coalescing events observed (Fig. 2D), including when normalised to mean Fascin bundle number (Fig. S1F).

Given their presence within the lamellipod and the suppressed migration of *ena* mutants, we next explored the relationship between Fascin bundle elongation/coalescence and motility. Tracking of the Ena–GFP cap on Fascin bundles revealed that the elongation rate of these bundles correlated positively with cell speed (Fig. 2E). The number of coalescing bundles/cell also increased with increasing speed (Fig. 2E).

In summary, we find Ena acts as a remodeller of lamellipodial actin, by firstly organising it into parallel aligned filaments cross-linked by Fascin, and secondly by mediating coalescence of these bundles into super-bundled structures. This remodelling of the lamellipod appears to be necessary for efficient cell migration.

### Ena expression compensates for loss of Fascin by promoting bundling within the lamellipod

**Fig. 3.**
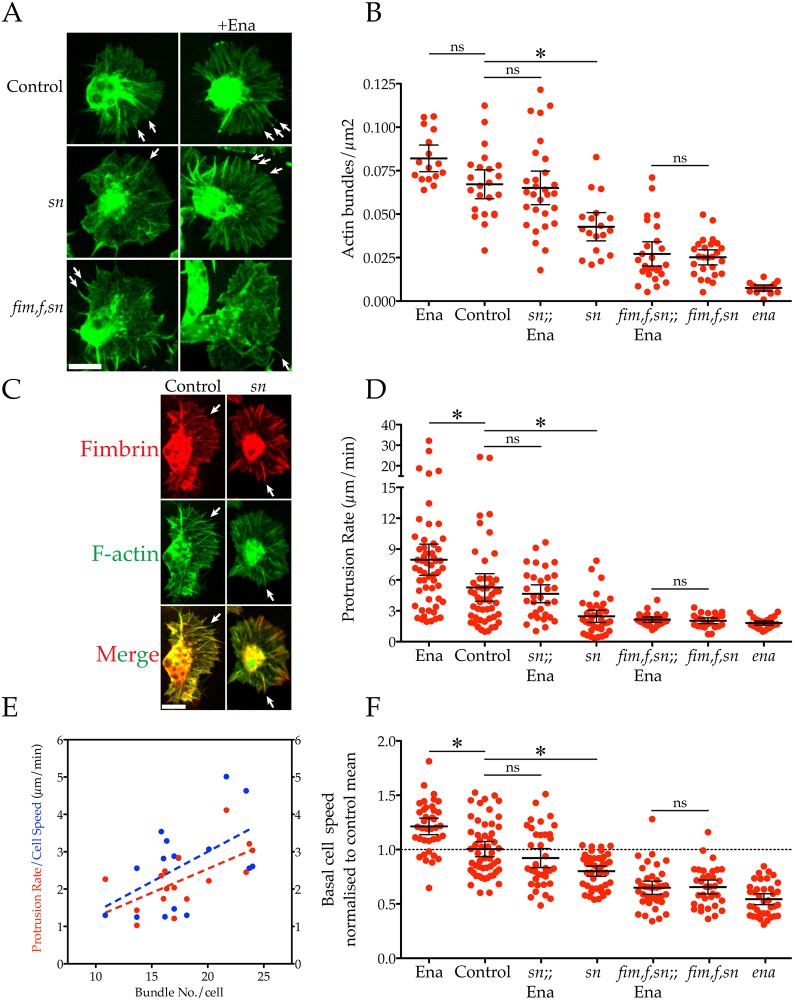
**Overexpression of Ena compensates for loss of Fascin by utilising the remaining actin bundlers to increase lamellipodial bundling, protrusion rate and basal speed.** (A) Control, *sn* or *fim, f, sn* (triple bundler mutant) macrophages expressing LifeAct–GFP with or without Ena–GFP. Arrows highlight bundles. Scale bar: 10 µm. (B) Quantification of actin bundle density within the lamellipods of control, *sn*, *fim*, *f*, *sn* and *ena* macrophages with or without Ena. Ena overexpression fails to increase bundling in *fim*, *f*, *sn* mutants (Ena=0.082±0.004, control=0.067±0.004, *sn*;; Ena=0.065±0.005, *sn*=0.043±0.004, *fim, f sn*;; Ena=0.027±0.003, *fim, f sn*=0.025±0.002, *ena*=0.008±0.001 bundles/µm^2^, mean±s.e.m., *n*≥15 cells/genotype). Error bars are 95% c.i. *P<0.05 vs control mean; ns, not significant (*P*>0.05) (ANOVA). (C) Colocalisation of fimbrin–mCherry (red) with LifeAct–GFP (green) at lamellipodia bundles (arrows) in control/*sn* mutants. Scale bar: 10 µm. (D) Quantification of lamellipodia protrusion rate of control, *sn*, *fim*, *f*, *sn* and *ena* macrophages with or without Ena. Ena overexpression fails to increase protrusion rate in *fim*, *f*, *sn* mutants (Ena=7.967±0.758, control=5.273±0.667, *sn*;; Ena=4.653±0.425, *sn*=2.461±0.295, *fim, f sn*;; Ena=2.031±0.133, *fim, f sn*=2.149±0.127, *ena*=1.807±0.101 µm/min, mean±s.e.m., *n*≥25 cells/genotype). Error bars are 95% c.i. *P<0.05 vs control mean; ns, not significant (*P*>0.05) (ANOVA). (E) Control bundle number/cell correlates positively with lamellipodial protrusion rate (red, r^2^=0.39, *n*=16) and basal cell speed (blue, r^2^=0.29, *n*=16). Both slopes are significantly different from zero (*P*<0.05). (F) Quantification of basal cell speed of control, *sn*, *fim*, *f*, *sn* and *ena* macrophages with or without Ena normalised to control mean (dashed line). Ena overexpression fails to increase basal speed in *fim*, *f*, *sn* mutants (Ena=1.208±0.037, control=1.000±0.034, *sn*;; Ena=0.918±0.043, *sn*=0.798±0.023, *fim, f sn*;; Ena=0.654±0.030, *fim, f sn*=0.654±0.031, *ena*=0.541±0.025 µm/min, mean±s.e.m., *n*>35 cells/genotype). Error bars are 95% c.i. *P<0.05 vs control mean; ns, not significant (*P*>0.05) (ANOVA).

Given the dependence of Fascin on Ena for lamellipodial bundling, we next explored the activity of Ena in Fascin (*sn*) mutants. Although *sn* macrophages have a significantly reduced number of lamellipodial bundles compared to controls, this is not the severe loss observed in *ena* mutants (Fig. 3A,B; Movie 4; Zanet et al., 2009). Surprisingly, despite lacking Fascin and its bundling activity, overexpression of Ena in *sn* mutants significantly increased lamellipodial bundling, restoring bundle number to control levels (Fig. 3A,B; Movie 4). Like most organisms, flies possess additional parallel actin bundlers, including Fimbrin (Fim) and Forked (F). We reasoned that the other bundlers must be responsible for bundling in the absence of Fascin (Vignjevic et al., 2006). Consistent with this hypothesis, fimbrin–mCherry localised to the remaining lamellipodial bundles present in the *sn* mutant (Fig. 3C; Movie 5). Crucially, Ena overexpression failed to increase lamellipodial bundling in macrophages mutant for all three of these actin bundlers (*fim*, *f*, *sn*, Fig. 3A,B; Movie 4). From these data, we conclude that although the different actin bundlers can partially compensate for one another within the lamellipod, they all depend on Ena at the leading edge and at the tip of the nascent bundle.

We next sought to understand how bundle number affected lamellipodial dynamics and how this related to cell speed. We used kymography to analyse leading edge extension and found that the decreasing bundle number found across the different genotypes was mirrored by incremental decreases in lamellipod protrusion rates (Fig. 3D). The overexpression of Ena in a control or *sn* background increased or rescued the lamellipod protrusion rate respectively. However, again this effect depended on cells retaining some bundling capacity, as Ena overexpression failed to increase the suppressed lamellipod protrusion rate of *fim*, *f*, *sn* triple mutants (Fig. 3D). Furthermore, these differences in lamellipodial dynamics translated into discrete differences in basal cell speed (Fig. 3F). Again, the overexpression of Ena restored the basal speed of *sn*, but not *fim*, *f*, *sn* macrophage migration.

In summary, increasing lamellipodial bundling promotes greater leading edge extension, which in turn drives the cell forward faster during migration. Consistent with these findings, increased bundle number correlated with both increased protrusion rate and basal speed in control macrophages (Fig. 3E). From these data, we conclude that although the different actin cross-linkers can partially compensate for one another, they all depend on Ena at the leading edge and at the tip of the nascent bundle to co-ordinate remodelling of the lamellipod and support robust migration.

### Increased bundling induced by Ena overexpression improves *sn* basal motility and chemotaxis during inflammation

**Fig. 4.**
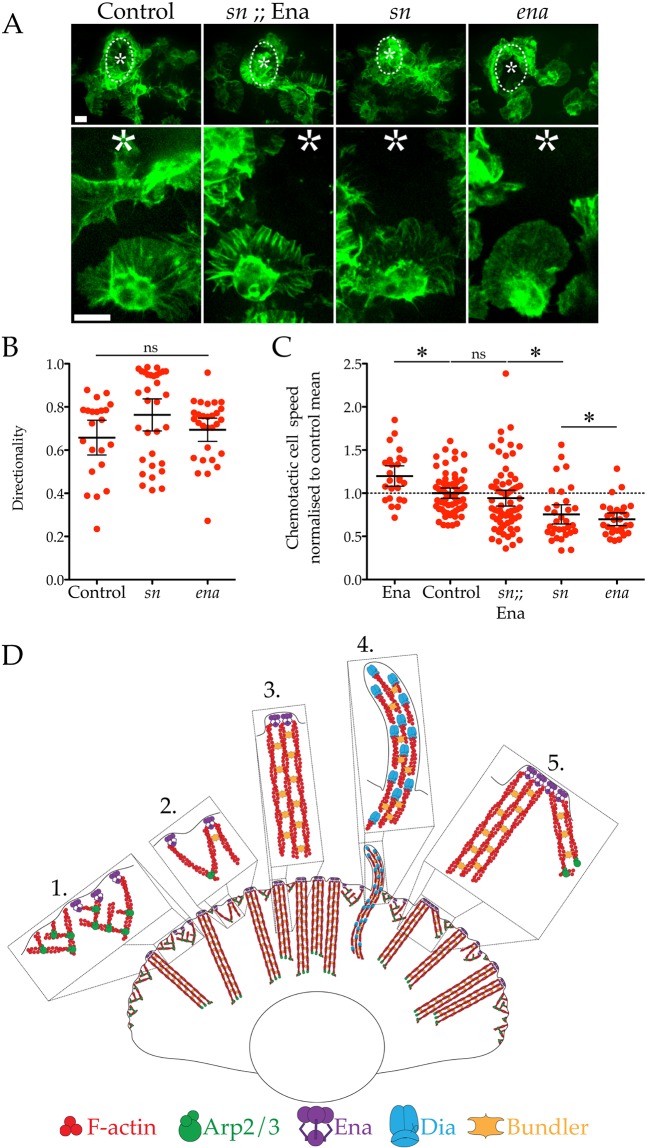
**Ena-mediated lamellipodial bundling drives robust recruitment during inflammation.** (A) Inflammatory response of control and *sn* macrophages with or without Ena–GFP, and *ena* macrophages recruited to laser-induced wounds (dashed outlines and asterisks). Top panels show low magnification of wounds at 10 min post ablation. Lower panels show cropped images of individual macrophages during inflammatory chemotaxis to wounds. Scale bars: 10 µm. (B) Quantification of control, *sn* and *ena* macrophage directionality during inflammatory chemotaxis. No significant differences were detected between any of the genotypes. ns, not significant (*P*>0.05) (ANOVA). (C) Quantification of control, *sn* and *ena* with or without Ena–GFP cell speed during inflammatory chemotaxis to wounds (Ena=1.198±0.057, control=1.000±0.030, *sn*;; Ena=0.943± 0.046, *sn*=0.754±0.055, *ena*=0.698±0.036 µm/min, mean± s.e.m., *n*≥25 cells/genotype). All values normalised to control mean (dashed line). *P<0.05 vs control mean; ns, not significant (*P*>0.05) (ANOVA). (D) Diagram highlighting the role of Ena in remodelling actin within lamellipodia. (1) The Arp2/3 complex (green) generates dendritic actin (red) in order to extend a lamellipodia. Ena (purple) at the leading edge captures growing filaments and elongates them linearly. (2) The unbranched, linear actin filaments (elongated and brought into proximity of each other by Ena) can now be cross-linked by bundlers such as Fascin and/or Fimbrin (orange). (3) Lamellipodial actin bundles cross-linked with Fascin or Fimbrin and capped by Ena act as struts to reinforce dynamic leading edge extensions, aiding cell migration. (4) In contrast, Dia (blue) generates distinct, Fascin cross-linked filopods. (5) Through multimerisation, Ena can coalesce lamellipodial bundles into super bundles. Ultimately, Ena remodels lamellipodial actin to promote efficient cell migration.

Given that Ena co-ordinates bundling within the lamellipod to promote efficient basal macrophage migration, we explored whether Ena also contributed to the inflammatory chemotaxis of macrophages towards wounds. We generated epithelial wounds through laser ablation and tracked macrophages during their recruitment (Fig. 4A). Neither *sn* or *ena* were required for macrophage recruitment to wounds and the directionality of these mutants during their inflammatory chemotaxis was indistinguishable from controls (Fig. 4A,B). However, loss of either *sn* or *ena* reduces macrophage speed towards such wounds (Fig. 4C; Zanet et al., 2009; Tucker et al., 2011). Again, we found that overexpression of Ena in *sn* mutants resulted in increased chemotactic speed and a more robust inflammatory response (Fig. 4C). Given the critical importance of rapid immune cell recruitment to sites of tissue injury, these data highlight the crucial role that Ena-generated actin bundles play in powering inflammatory chemotaxis *in vivo*.

Taking all these data together, we propose that Ena captures branched actin filaments generated by the Arp2/3 complex by binding their barbed-end and overseeing their continued elongation (Fig. 4D). Ena achieves this by both preventing barbed-end capping by capping protein and/or through the ability of Ena to processively elongate actin filaments (Bear et al., 2002; Winkelman et al., 2014). Once in control of filament elongation, Ena can bring together other similarly elongating filaments to be bundled by cross-linkers such as Fascin (Winkelman et al., 2014). Through the ability of constitutively tetrameric Ena to further multimerise, Ena can promote coalescing of bundles into higher-order bundled structures (Breitsprecher et al., 2008). Positioned perpendicular to the membrane, we propose that these lamellipodial bundles act as cytoskeletal struts, exerting maximum force on the leading edge and reinforcing it when it does protrude (Mueller et al., 2017).

We envision Ena acting at the leading edge to generate lamellipodial bundles via convergent elongation and ultimately remodelling dendritic actin into linear actin bundles (Svitkina et al., 2003). Furthermore, owing to its known physical interactions with other actin regulators such as the SCAR complex and Dia, it is perfectly placed to act as a master regulator of the cytoskeleton by co-ordinating nucleators, cross-linkers and F-actin itself (Chen et al., 2014; Bilancia et al., 2014; Schirenbeck et al., 2006).

### Concluding remarks

Since both Ena and Dia generate actin bundles, disentangling their activities from one another is challenging, especially since mammals possess multiple homologs of both. As demonstrated here, *Drosophila* macrophages are skewed towards high Ena rather than Dia activity, possibly maintained through negative regulation of Dia mediated by Ena (Bilancia et al., 2014). This has yielded a unique opportunity to clarify their roles, even when compared to other motile cells within *Drosophila* that exhibit a blend of Ena and Dia activity (Homem and Peifer, 2009; Nowotarski et al., 2014).

Here, we demonstrated that Ena acts to remodel F-actin within the lamellipod into Fascin-decorated bundles. We do not mean to dismiss the well-established role of Ena as a nucleator. In different cells, Ena does generate filopods *de novo* (Rotty et al., 2014). However, in highly motile cells, which are migrating through the use of broad lamellipods, we propose Ena primarily functions as a remodeller of dendritic actin to promote formation and elongation of lamellipodial bundles. In this role, Ena acts to marshall actin and the other actin regulators within the lamellipod in order to co-ordinate the cytoskeleton during critical processes, such as the inflammatory recruitment of macrophages to wounds.

How macrophages couple the recognition of inflammatory stimuli to rearrangements in the actin cytoskeleton remains poorly understood. In *Drosophila*, the immunoreceptor tyrosine-based activation motif (ITAM)-containing MEGF10 homolog, Draper, has a central role in relaying the detection of H_2_O_2_ released upon wounding to the Syk-family kinase Shark (Evans et al., 2015). However, exactly how this signalling feeds down to the Rho-family GTPases and actin regulators such as Ena, which are driving chemotaxis, is not known.

Further studies are required to bridge the gap between the signals that guide macrophages and the cytoskeletal regulators that power their motility. However, from this study it is clear that Ena is a master remodeller within the lamellipod, allowing macrophages to harness the full force of the actin cytoskeleton during inflammatory chemotaxis where the rapidity of this response determines survival of the organism as a whole.

## MATERIALS AND METHODS

### Fly stocks

*SingedGAL4* (*sn-GAL4*, Zanet et al., 2012) was combined with *serpentHemoGAL4* (*srp-GAL4*, Brückner et al., 2004) and *croquemortGAL4* (*crq-GAL4*, Stramer et al., 2005) to drive expression of UAS constructs specifically in hemocytes. The following UAS constructs were used in this study: *UAS-GFP-Ena*, *UAS-FPPPPmito-GFP* (Gates et al., 2007), *UAS-mCherry-Fascin* (Zanet et al., 2009), *UAS-LifeAct-GFP* (Hatan et al., 2011)*, UAS-Dia*Δ*DAD-GFP* (Homem and Peifer, 2009) and *UAS-Dia-GFP* (Homem and Peifer, 2008). *UAS-fimbrin-mCherry* was generated in-house. *UAS-LifeAct-mCherry* flies were generated by introducing sequence encoding *LifeAct-mCherry* into pATTB-UASt, which was then sent for commercial injection (Best Gene Inc). The amorphic mutant alleles used in this study were: *arp3[EP3640]* (Hudson and Cooley, 2002), *dia[2]*, *dia[5]* (Castrillon and Wasserman, 1994), *ena[GC1]* (Gertler et al., 1995), *sn[28]* (Cant and Cooley, 1996) and *scar[37]* (Zallen et al., 2002). *dia[2]/dia[5]* maternally zygotic embryos were generated as in Homem and Peifer, 2008. A *fimbrin*, *forked*, *singed* triple mutant was generated by recombining *Df(1)BSC584* (Bloomington) with *sn[28]* in-house.

### Embryo genotypes

A minimum of three embryos/genotype were imaged in every case. For assessing the colocalisation of actin regulators: (1); sn-Gal4, UAS-arp3-gfp; sn-Gal4, UAS-lifeact-mcherry, (2); sn-Gal4, UAS-lifeact-mcherry; crq-Gal4, UAS-ena-gfp, (3); sn-Gal4, UAS-lifeact-mcherry; UAS-diaΔdad-gfp, (4); sn-Gal4, UAS-dia-gfp; sn-Gal4, UAS-lifeact-mcherry, (5); ena[GC1], sn-Gal4, UAS-dia-gfp; sn-Gal4, UAS-lifeact-mcherry, (6); srp-Gal4, UAS-fimbrin-mcherry; sn-Gal4, UAS-lifeact-gfp and (7) sn[28], sn-Gal4, UAS-lifeact-gfp; srp-Gal4, UAS- fimbrin-mcherry. For the imaging and quantification of actin bundles: (1); sn-Gal4, UAS-lifeact-gfp, (2); ena[GC1], sn-Gal4, UAS-lifeact-gfp, (3); dia[2], sn-Gal4, UAS-lifeact-gfp, (4) dia[2], sn-Gal4, UAS-lifeact-gfp/dia[5], (5); scar[37], sn-Gal4, UAS-lifeact-gfp, (6);; arp3[EP3640], sn-Gal4, UAS-lifeact-gfp, (7); sn-Gal4, UAS-lifeact-gfp; crq-Gal4, UAS-ena-gfp, (8) sn[28], sn-Gal4, UAS-lifeact-gfp, (9) sn[28], sn-Gal4, UAS-lifeact-gfp; crq-Gal4, UAS-ena-gfp, (10) df(1)BSC584, sn[28]; sn-Gal4, UAS-lifeact-gfp, (11) df(1)BSC584, sn[28]; sn-Gal4, UAS-lifeact-gfp; crq-Gal4, UAS-ena-gfp, (12); sn-Gal4, UAS-lifeact-gfp; UAS-diaΔdad-gfp, (13); ena[GC1], sn-Gal4, UAS-lifeact-gfp; UAS- diaΔdad-gfp and (14); ena[GC1], sn-Gal4, UAS-lifeact-gfp; crq-Gal4, UAS-ena- gfp were used. For the imaging and quantification of Fascin bundles: (1) sn[28], sn-Gal4, UAS-sn-mcherry; ena[GC1], (2) sn-Gal4, UAS-sn-mcherry, (3) sn[28], sn-Gal4, UAS-sn-mcherry;; crq-Gal4, UAS-ena-gfp and (4) sn[28], sn-Gal4, UAS-sn-mcherry; sn-Gal4, UAS-dia-gfp were used.

### Live imaging

Developmental stage 15 embryos were collected in cell strainers (Falcon), dechorionated with bleach (Jangro), washed vigorously with water and mounted between a glass slide and a supported coverslip in droplets of VOLTALEF oil (VWR) as previously described (Evans et al., 2010). Ventral hemocytes were then imaged using a spinning disc confocal microscope (Perkin Elmer Ultraview) with a plan-apochromat 63× objective with a NA of 1.4 and a Hamamatsu C9100-14 camera. The acquisition software used was Volocity (Perkin Elmer). Epithelial wounds were generated using laser ablation (nitrogen-pumped micropoint ablation laser tuned to 435 nm, Andor Technologies) as previously described (Wood et al., 2002).

### Image processing and statistical analysis

All acquired images were imported into ImageJ (NIH) and maximally projected. Lamellipods were outlined by hand to measure their area (excluding cell body). Actin bundles were defined as any linear concentration of LifeAct–GFP and counted manually. In both cases, cell means were derived from the analysis of all frames between cell–cell collisions. Cell speed was derived from tracks generated using the ImageJ manual tracking plugin (severely enlarged *dia* M/Z macrophages were excluded from cell speed analysis). Kymographs were constructed using the ImageJ reslice tool and used to calculate leading edge protrusion rates. Fascin bundles were defined as any linear concentration of Fascin–mCherry and were counted manually. Bundle tips were tracked during extension to derive bundle protrusion rates and capture coalescence events. Coalescence was defined as the sustained alignment (>2 frames, 12 s) of two or more bundles per minute. Unpaired, two-tailed *t*-tests and one-way ANOVA with a Tukey's multiple comparisons test were used to test statistical significance and generate *P* values.

## Supplementary Material

Supplementary information
