# Supplementary material for: Ena orchestrates remodelling within the actin cytoskeleton to drive robust Drosophila macrophage chemotaxis
Source: J Cell Sci. 2019 Feb 18;132(5):jcs224618. doi: 10.1242/jcs.224618 (PMC6432709; doi:10.1242/jcs.224618)
Supplement: Supplementary information [file joces-132-224618-s1.pdf]

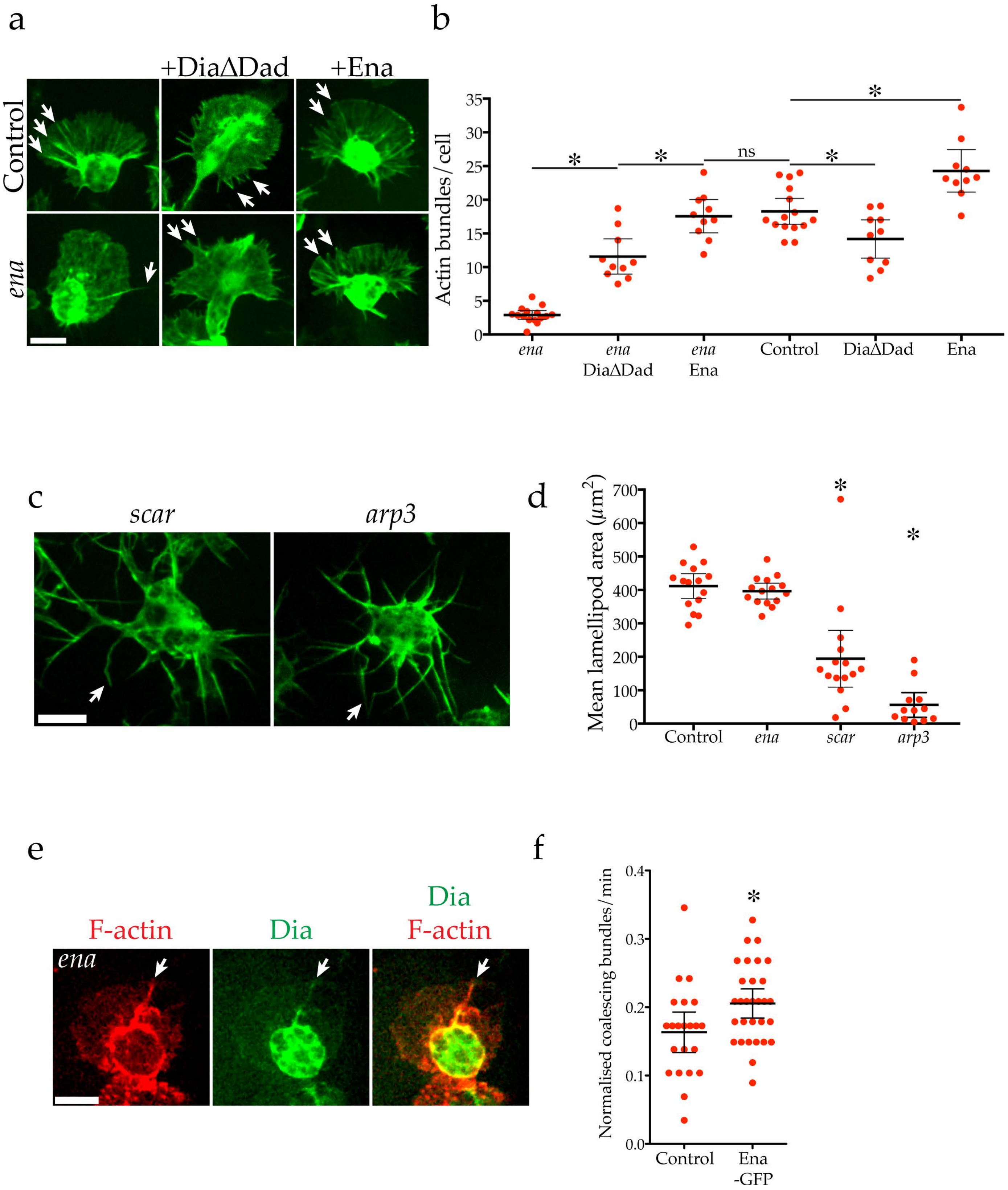

Journal of Cell Science • Supplementary information

Figure S1. Ena remodels the lamellipod into fascin-decorated, actin-bundles

(a) control or *ena* macrophages expressing LifeAct-GFP and either DiaΔDad-GFP or Ena-GFP. Arrows highlight actin bundles. Scale bar =10 μm.

(b) Actin bundles/cell in control or *ena* macrophages expressing either DiaΔDAD-GFP or Ena-GFP (*ena* =2.90 ±0.31, *ena*; DiaΔDAD =1.58 ±1.16, *ena;* Ena =17.56 ±1.09, control =18.28 ±0.90, DiaΔDAD =14.18 ±1.26, Ena =24.28 ±1.39 bundles/cell, mean ±SEM, n ≥10). Error bars are 95% CI and asterisks indicate statistical significance (ANOVA, $p<0.05$). ns =$p>0.05$.

(c) *scar* and *arp*3 mutants expressing LifeAct-GFP (GREEN). Arrows highlight actin bundles/filopods. Scale bar =10 μm.

(d) Mean lamellipod area of control, *ena, scar* and *arp3* macrophages (control =411.68 ±17.20, *ena* =396.57 ±11.04, *scar* =194.41 ±39.64, *arp3* =56.05 ±16.91 μm^2, mean ±SEM, n ≥12 cells/genotype).

(e) Colocalisation of LifeAct-mCherry (RED) with Dia-GFP (GREEN) at the residual lamellipodial bundles (arrows) in *ena* mutants. Scale bar =10 μm.

(f) Number of fascin bundle coalescence events/min normalised to mean fascin bundle number of each genotype (control = 0.1634 ±0.014, Ena =0.206 ±0.010 events/cell, mean ±SEM, n >20 cells/genotype). Error bars are 95% CI and asterisks indicate statistical significance vs. control mean (unpaired t test, $p<0.05$).

Journal of Cell Science • Supplementary information

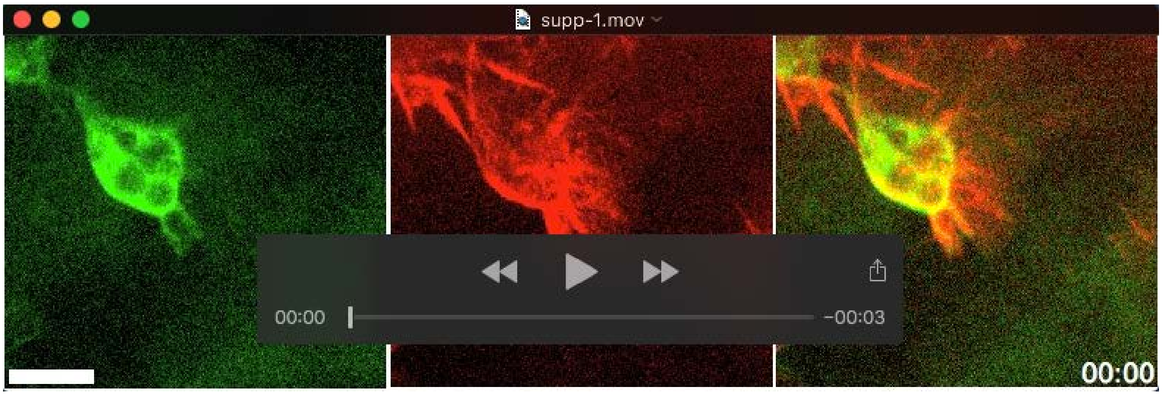

Movie 1. ***In vivo*** **localisation of Dia in motile** ***Drosophila*** **macrophages.** Full length Dia-GFP (GREEN) localises to rare actin bundles (LifeAct-mCherry, RED) within the lamellipod. Images were acquired with spinning disc confocal microscopy (Perkin Elmer Ultraview) every 30 s. The scale bar is 10 μm. The movie frame rate is 4 frames/s.

Journal of Cell Science • Supplementary information

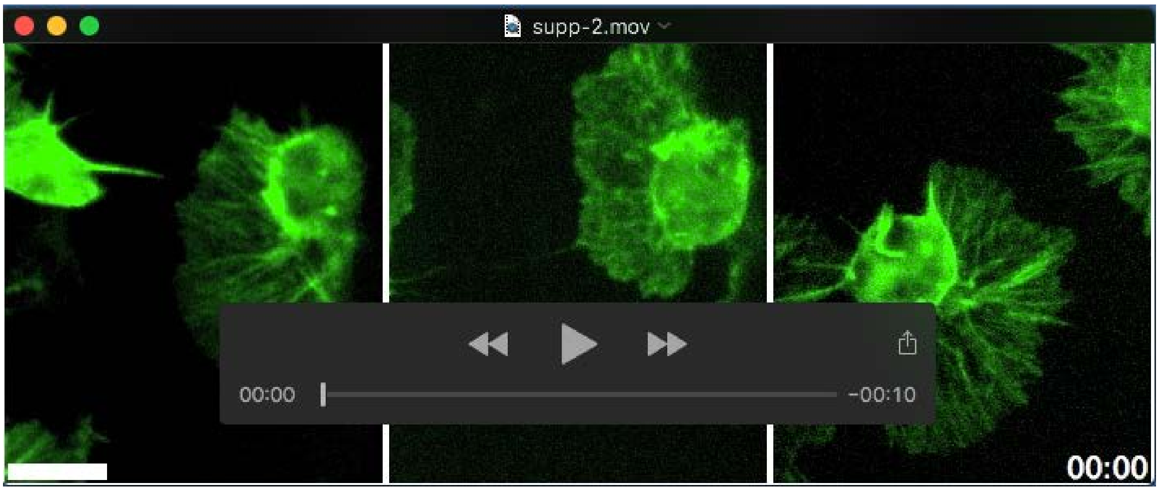

Movie 2. Loss of *ena* but not *dia* results in loss of nearly all lamellipodial bundles. Lamellipodial bundling in control, *ena* and *dia* M/Z macrophages expressing LifeAct-GFP (GREEN). A rare, probing *ena* actin bundle/filopod is shown. Images were acquired with spinning disc confocal microscopy (Perkin Elmer Ultraview) every 30 s. The scale bar is 10 μm. The movie frame rate is 4 frames/s.

Journal of Cell Science • Supplementary information

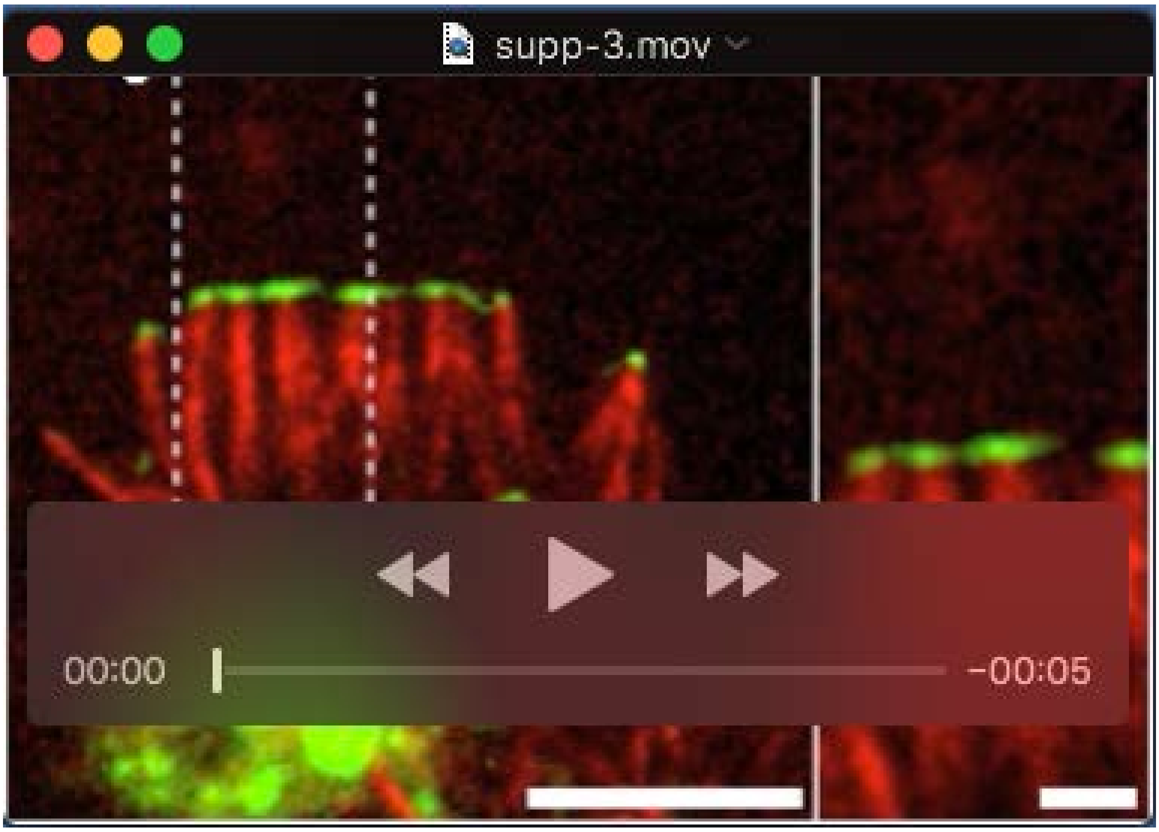

Movie 3. Ena mediates coalescence of fascin bundles. Examples of Ena-GFP (GREEN) capped fascin bundles (Fascin-mCherry, RED) coalescing within a protruding lamellipod. Dashed box is enlarged in left-hand panel. Images were acquired with spinning disc confocal microscopy (Perkin Elmer Ultraview) every 6 s. Time is in seconds and the scale bars =10/2 μm. The movie frame rate is 4 frames/s.

Journal of Cell Science • Supplementary information

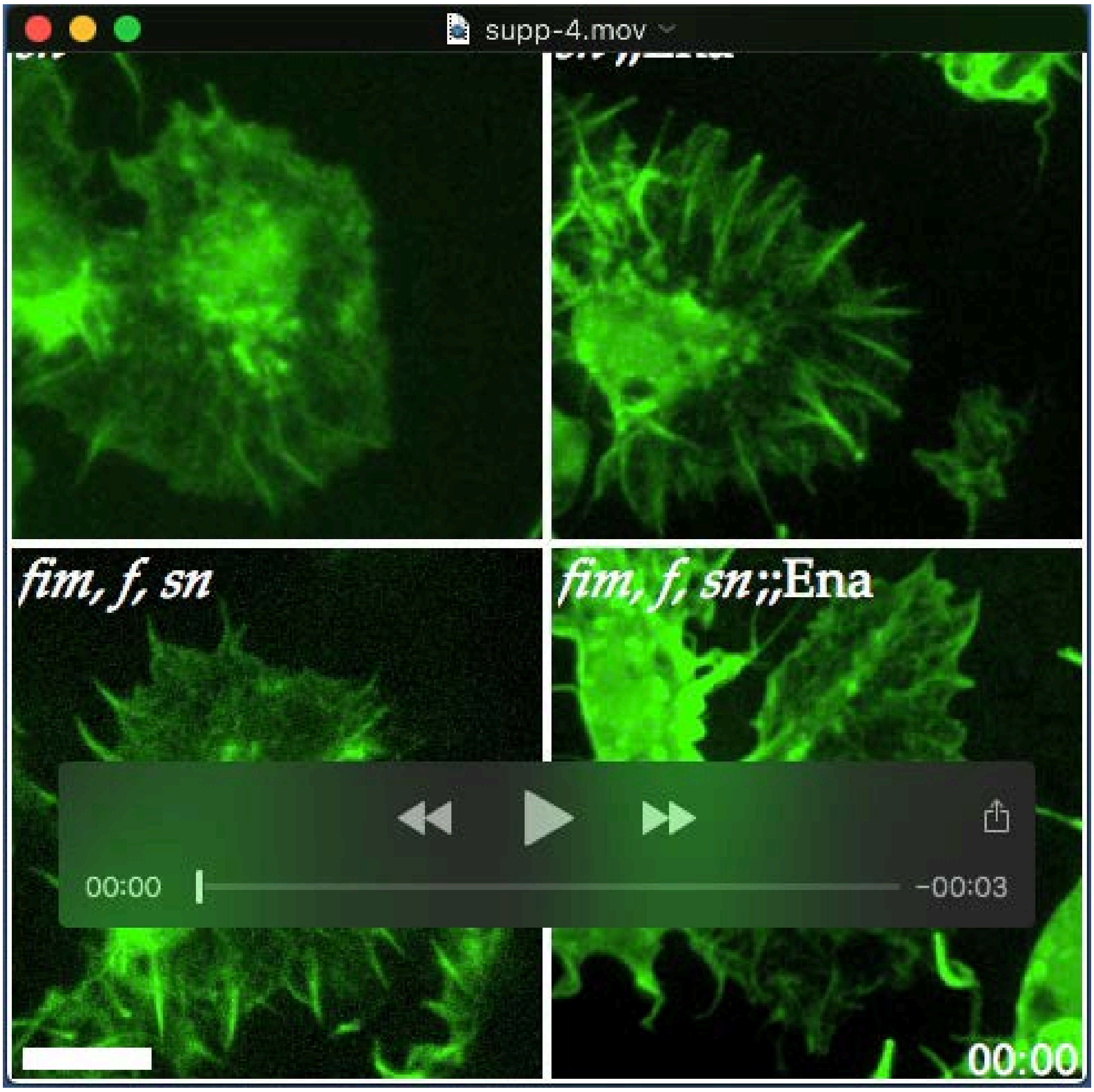

Movie 4. Ena over-expression utilises fimbrin and/or forked to restore lamellipodial bundling in absence of fascin. Actin bundling (LifeAct-GFP, GREEN) in *sn* or *fim, f, sn* triple mutants ±Ena over-expression. Images were acquired with spinning disc confocal microscopy (Perkin Elmer Ultraview) every 30 s. The scale bar is 10 μm. The movie frame rate is 4 frames/s.

Journal of Cell Science • Supplementary information

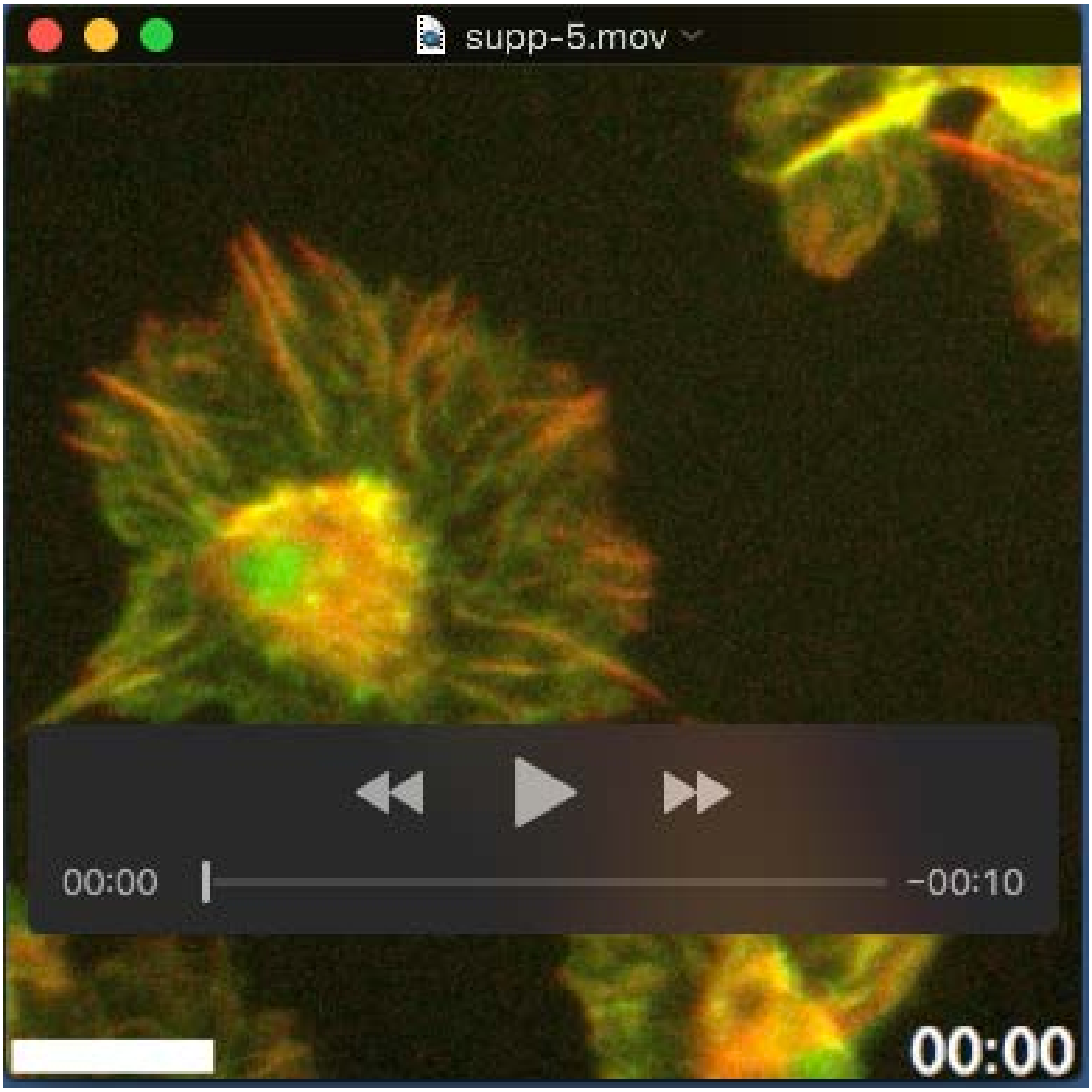

Movie 5. Fimbrin compensates for loss of fascin to maintain lamellipodial bundling. Fimbrin-mCherry (RED) decorates actin bundles (LifeAct-GFP, GREEN) within the lamellipod of *sn* mutant macrophages. Images were acquired with spinning disc confocal microscopy (Perkin Elmer Ultraview) every 30 s. The scale bar is 10 μm. The movie frame rate is 4 frames/s.

Journal of Cell Science • Supplementary information
